# Free Your Mind: Creative Thinking Contributes to Overcoming Conflict-Related Biases

**DOI:** 10.3390/brainsci12111566

**Published:** 2022-11-17

**Authors:** Nardine Fahoum, Hadas Pick, Tal Ivancovsky, Simone Shamay-Tsoory

**Affiliations:** 1 Department of Psychology, University of Haifa, Haifa 3498838, Israel; 2 The Gonda Multidisciplinary Brain Research Center, Bar Ilan University, Ramat-Gan 5290002, Israel

**Keywords:** divergent thinking, intergroup conflicts, default mode network, executive control network

## Abstract

Conflicts between groups are difficult to resolve, partly because humans tend to be biased in judging outgroup members. The aim of the current article is to review findings on the link between creativity and conflict-related biases and to offer a model that views creative cognition as an ability that may contribute to overcoming conflict-related biases. Our proposed model conforms to the twofold model of creativity. According to this model, creativity involves a generation phase and an evaluation phase, and these phases correspond to the neural mechanisms that underlie conflict-related biases. Specifically, we contend that the generation phase of creativity affects conflict-related biases by exerting an influence on stereotypes and prejudice, outgroup-targeted emotions, and ingroup empathy biases, all of which rely on the default mode network. Conversely, the evaluation phase of creativity, which is usually associated with activation in the executive control network and action-observation system, may be related to herding behaviors. Building on the shared mechanisms of creativity and conflicts, we propose that studies examining creativity-based interventions may be effective in promoting reconciliation.

## 1. Introduction

Intergroup conflicts vary from tractable to intractable conflicts and from conflicts between ethnic or religious groups to those between states [1]. The consequences of violent conflicts are fatal and account for millions of deaths [2]. Therefore, understanding the mechanisms underlying intergroup conflicts may likely hold the key to enhancing reconciliation and peacemaking.

Developing interventions aimed at promoting coexistence between groups is extremely challenging. Much research on interventions for conflict reduction has focused on contact between parties [3], peace education [4], and perspective-taking [5]. While these interventions have proven to be effective, some appear to be ineffective for members of the non-dominant group [6], and others are ineffective in active real-life conflicts [7]. Moreover, not many of these interventions focused on cognitive abilities that have the potential to overcome intergroup biases. Here we suggest that focusing on creative cognition may offer a new avenue for interventions aimed at diminishing group-related biases. To this aim, the manuscript synthesizes findings about creativity with those on conflict-related biases and proposes a link between these seemingly different behaviors. The article begins with an explanation of our method. Then, we define creativity and discuss the two-fold model of creativity, including the brain mechanisms that underlie it. We continue by describing the potential role of creativity as an intervention and reviewing components of creativity that might help in overcoming conflict-related biases. We then focus on four conflict-related biases: stereotypes and prejudice, outgroup-targeted emotions, ingroup empathy biases, and herding. We define these biases and discuss brain regions that support these biases, review studies linking them to creativity, and argue that a common set of brain networks underlies both creativity and cognitive-related biases. We continue by proposing creativity-based interventions and providing directions for future studies. Finally, we discuss the limitations and challenges of our study and model and conclude that creativity overlaps with many mechanisms that may be important in reducing conflict-related cognitive biases, among them prejudice, negative emotions, and lack of empathy toward outgroup members.

## 2. Methods

The aim of the current review is to collect indirect evidence for the shared mechanisms between creativity and conflict-related biases and to demonstrate the existence of a link between creativity and cognitive-related biases. The approach that we employed is a non-systematic narrative review. The best approach is a non-systematic narrative review [8] because this review discusses a concept that is in progress and not an established one.

## 3. Creativity: Definition and Brain Models

Creativity is defined as the ability to produce novel and useful ideas [9]. Being creative influences our achievements in various domains, from the invention of new technologies that facilitate our everyday life to the development of artistic output for our pleasure and entertainment. The houses we live in, the cars we drive, and the clothes we wear are all products of human inventiveness [10]. Creative ideas come from novel viewpoints offered by individuals in a specific situation [11].

Traditional approaches to creativity contend that creative thinking involves the arrangement of associative elements into new and useful combinations [12]. This process comprises two stages: idea generation, in which ideas are brought together in unusual ways to create an original product, and evaluation of these ideas for their appropriateness and novelty [9]. Building on this view, which is also known as the Two-Fold Model of Creativity, neuroimaging studies that examine the neural underpinnings of creativity link the default mode network (DMN) to creative thinking, and particularly to the generation phase of the creative process [13,14,15]. The DMN is a network of brain regions originally identified in functional magnetic resonance imaging (fMRI) studies during task-free trials [16]. It consists of the anterior cingulate cortex (ACC), the posterior cingulate cortex (PCC), the medial prefrontal cortex (mPFC), and the temporo-parietal junction (TPJ) [17,18]. Activity in the DMN is frequently associated with spontaneous cognitions and self-generated thought, including mind wandering, future thinking, memory retrieval, and divergent thinking [19,20,21,22]. Divergent thinking tasks, which measure the ability to come up with multiple solutions to an open problem [23,24], are considered valid tools for examining creative ability [17,25,26]. The DMN was found to be strongly activated among creative individuals, and this activation was associated with higher originality scores on divergent thinking tasks [13,27].

While the DMN was found to contribute to the generation phase of creativity, the executive control network (ECN) has been suggested as participating in the evaluation phase. The ECN is associated with cognitive processes grounded in different prefrontal regions, including the inferior frontal gyrus (IFG) and the dorsolateral prefrontal cortex (dlPFC) [28]. Evidence from neuroimaging and lesion studies suggests that damage to the left IFG, which causes reduced inhibitory control, leads to increased creative production [26,29]. Moreover, patients with frontotemporal dementia, which is characterized by damage in the left IFG, exhibit enhanced artistic creativity [30,31,32]. Further evidence from transcranial magnetic stimulation (TMS) studies demonstrated that temporary inhibition of the left IFG leads to higher originality scores as a result of less stringent evaluations [33]. Considering that the left IFG was shown to be active during idea evaluation [26,34,35], reduced activity in this region may, in fact, lead to less strict evaluations and, consequently, to increased creativity. These ideas are in line with a study suggesting that while right mPFC lesions were found to be associated with impaired creativity scores, patients with left IFG lesions exhibited high creativity scores [29].

Both the generation and the evaluation phases seem to be involved in overcoming stereotypes. Stereotypes represent an associated network that includes automatic and close associations, where the activation of one node automatically activates related nodes; for example, mechanic–male [36,37]. In order to overcome stereotypes, it is essential to form new and remote associations, an ability that involves the generation phase [12]. Given that this phase is associated with internally oriented cognition [22], it may contribute to experiencing emotions in a non-automatic way that is not influenced by external factors (e.g., experiencing more empathy toward the outgroup during conflicts instead of anger). It may also help in bringing ideas together in unusual ways to create a new and original perspective about the outgroup that may result in diminished biases. In addition, less strict evaluations of these associations are crucial for a mindset that involves openness and acceptance. Therefore, we expect that an increase in the generation phase and a decrease in the evaluation phase might contribute to reducing intergroup biases.

## 4. Creativity as an Intervention

One key component of creativity that may be important in the relationship between creativity and conflict resolution is flexible thinking. Cognitive flexibility is related to the ability to break old cognitive patterns, overcome functional fixedness [23] and switch from one perspective to another [38]. The flexibility of thought is essential for creativity as it allows for frequent switches among categories during the creative process [39] and, as a result, generates new associations between different concepts. Indeed, highly creative individuals were found to be more flexible in their thinking [40,41]. Furthermore, flexibility is essential for problem-solving in that it facilitates the ability to focus attention selectively, inhibits extraneous information, and allows for flexible shifts in attention across multiple elements during the process [22,42,43]. In the context of conflict resolution, cognitive flexibility may contribute to the ability to overcome automatic cognitive biases and to more easily generate creative solutions to the situation. Indeed, resolving a conflict in a constructive and cooperative manner facilitates the ability to recognize contradictions and maintain flexibility in processing contradictory information or viewpoints [44,45,46].

Another component of creativity that may affect intergroup biases and conflicts is originality, defined as the generation of associations that are uncommon, remote, infrequent, and rare relative to existing ones [23,47]. This component may contribute to experiencing emotions that differ from automatic outgroup-targeted emotions. Additionally, creativity is positively associated with openness to experience [48], an ability that may facilitate experiencing novel and remote emotions. Greater openness may help in being able to explore new attitudes and emotions. In support of our hypothesis, studies have shown that people high in openness experience a broader range of emotions than people low in openness [49]. In line with this, open-minded individuals exhibit greater cognitive flexibility during conflict resolution, as they tend to use a wider range of ideas and more diverse strategies [50]. Interestingly, implicit theories within a conflict [51,52] suggest that individuals who are flexible in the belief that the outgroups’ traits could change are more open to negotiation [53] and to cooperation [54] and experience more positive attitudes toward the outgroup [55]. One study manipulated the belief about the outgroup’s changeability and found that participants in the changeable condition reported lower levels of anxiety and more willingness for intergroup contact than participants who believed that the outgroups are fixed [56]. Specifically, anxiety mediated the causal effect of changeability on the desire for contact. Given that hope is associated with cognitive flexibility [57], another study examined the role of hope in the effect of the changeability of a conflict on compromise in conflict. The findings indicate that changeability belief led to more compromise through the process of increased hope [58]. These findings suggest that during conflicts, people tend to have a rigid mindset [59,60,61] that affects emotions and attitudes experienced toward the outgroup and the conflict. Thus, training the mind to think more creatively and flexibly might help in experiencing more positive emotions and attitudes during conflicts.

Based on the above, components of creativity, including openness, flexibility, and originality, may possibly influence intergroup conflict by affecting conflict-related factors. Cognitive biases represent a systematic pattern of deviation from rational judgment. Here we focus on four conflict-related biases that are potentially modulated by creativity: stereotypes and prejudice, outgroup-targeted emotions, ingroup empathy biases, and herding.

## 5. Stereotypes and Prejudice

Stereotypes refer to the preexisting categorical beliefs and attributes linked to a group [62,63]. In contrast, prejudice consists of attitudes and emotional responses toward a group [64]. Stereotypes and prejudice are manifested in routine social life. For instance, after terrorist bombings in Israel, Jewish judges tend to favor Jewish plaintiffs in their judicial decisions, while Arab judges tend to favor Arab plaintiffs [65]. Moreover, stereotypes and prejudice are apparent at an early age and have many mental and physical health consequences for group members [66,67]. Fiske [68] suggested that stereotypes and prejudice underlie aggressive behavior and are the core precursor of intergroup conflicts.

Even though prejudice and stereotyping are conceptually distinct, the brain structures involved in the two overlap, as social behavior is affected by an amalgamation of both functions [69]. One of these structures is the mPFC, which is implicated in the processing of social information and in forming impressions of others [70,71]. Deactivation of the mPFC was found during tasks involving prejudice and stereotyping [72,73]. Indeed, Sellaro et al. [74] pointed out the causal role of the mPFC in stereotype activation. In their experiment, they administered transcranial direct current stimulation (tDCS) to the mPFC while participants performed the Implicit Association Test (IAT), a well-established test that measures stereotypes and biases. The researchers found that IAT scores were reduced after anodal (excitability) stimulation, suggesting that increased activity in the mPFC decreases stereotypes and prejudice. Thus, enhanced activation of the mPFC may have the potential to reduce stereotypes and prejudice. Given that the generation phase of creativity is associated with increased activation in the mPFC [14], it appears that such activation may be able to overcome stereotypes and prejudice.

One aspect of creativity that may be related to stereotypes and prejudice is the breadth of thought and the flexible semantic network structure [75]. According to the associative theory of creativity, creative individuals tend to exhibit a richer and more flexible associative network than less creative individuals [12]. Recent studies demonstrate that creative thinkers tend to have a flexible semantic network, which is marked by shorter path lengths (smaller distances between concepts with fewer mediating associations) and increased interconnectivity between concepts [40,76,77,78,79]. Short path lengths are indicative of faster diffusion of information and smaller distances between concepts with fewer mediating associations [80]. For example, Gray et al. [77] showed that highly creative individuals can search farther along their semantic network and retrieve more remote free associations. Thus, for highly creative people, more distant concepts seem to appear closer in their semantic networks. Stereotypes and prejudices are examples of less flexible semantic networks [81,82,83]. Therefore, the intervention that involves training the ability to search for distant associations may help encourage the generation of new associations related to conflict biases.

Note that creativity was also found to be linked to stereotyping and prejudice in studies that demonstrated an improvement in creativity following counter-stereotypic interventions. These interventions were found to reduce stereotypes and prejudice [84] and to require thinking contrary to stereotypic expectations [81,85]. For example, Gocłowska and Crisp [85] showed participants either a stereotypic target, such as a male mechanic, or a counter-stereotypic target, such as a female mechanic. They then measured creativity performance using the alternate uses task—a divergent thinking task. More original and flexible responses emerged in the counter-stereotypic condition than in the stereotypic condition only among participants who scored low on a scale measuring the tendency to use mental representations such as schemata, indicating that this intervention is useful for certain individuals. Groyecka [86] previously discussed the opposite direction of this relationship, i.e., the effect of creativity on stereotypes. She proposed that creative thinking training may be used as an intervention to reduce stereotyping through three abilities related both to creative thinking and to stereotypes: cognitive flexibility, openness to experience, and perspective-taking. In line with this view, in Groyecka-Bernard et al. [87], participants underwent two creativity manipulations in two different experiments. The first creativity training was an imagination task in which the participants were asked to describe what they saw on an unfamiliar and distant planet where they had just landed. The second training was a divergent thinking task in which they were asked to generate five titles for two images. After both creativity interventions, participants scored lower on prejudice measures than controls.

**Figure 1:**
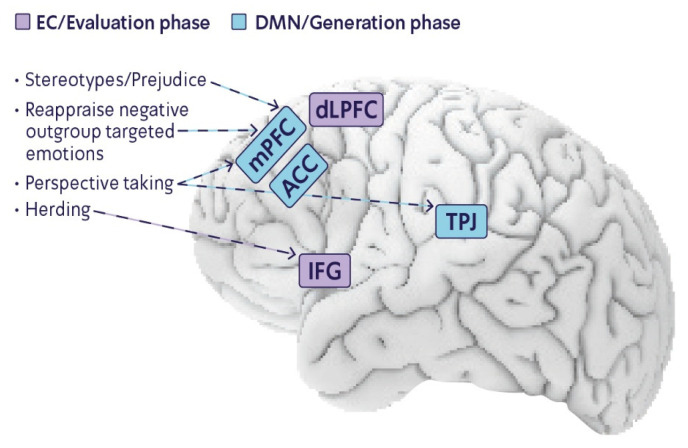
Common set of brain networks underlies both creativity and cognitive-related biases. The generation phase (blue) comprises the mPFC, ACC, and TPJ, which also involve intergroup biases. The evaluation phase (purple) comprises the IFG, which also involves herding.

To conclude, the studies discussed above show that creativity intervention may involve an increase in activations of the mPFC, which is associated with reduced stereotypes and prejudice (see Figure 1). Creativity encourages a broad thinking style in which the distance between remote concepts decreases, thus diminishing stereotypes and prejudices that involve the activation of close automatic associations.

## 6. Outgroup-Targeted Emotions

Negative outgroup-targeted emotions are emotions shared by group members that target outgroup members [88]. Outgroup-targeted emotions may include anger, hate, and fear and are often experienced during conflicts [1,89,90]. These emotions were found to affect conflict-related attitudes (e.g., [91]). For example, Porat et al. [92] found that emotion regulation intervention decreases aggressive conflict-related attitudes by means of a decrease in negative outgroup-targeted emotions. Experiencing outgroup-targeted emotions in more moderated and regulated ways that are far from the automatic emotions usually experienced may change conflict-related attitudes and consequently lead to peace.

Extensive research has examined the effect of cognitive reappraisal on outgroup-targeted emotions [92,93,94]. Cognitive reappraisal is an emotion regulation strategy that involves altering the biased interpretation of a situation and consequently changing the emotions elicited by it [95]. For example, Halperin et al. [96] found that Israelis who were asked to reappraise their emotions while reading an anger-provoking text reported higher levels of positive emotions and lower levels of negative emotions toward Palestinians. Literature on the neural correlates of cognitive reappraisal suggests that increased activity in the mPFC underlies the regulation of negative emotions [97]. For example, increased activation in the mPFC has repeatedly been found in experiments involving reappraisal conditions in which participants are shown negative emotional pictures [98]. In this regard, Weber et al. [99] found that the Reappraisal Inventiveness Test, which measures the ability to generate a different interpretation for a situation that provokes anger, is positively related to divergent thinking and openness to experience. Thus, creativity may help in emotion regulation, which in turn helps diminish negative emotions toward outgroup members. 

To examine the relationship between creativity and conflict related emotions, a recent study assessed originality as measured by the Torrance Test of Creative Thinking [24], outgroup-targeted emotions, and attitudes toward the conflict in the context of the Israeli–Palestinian conflict [100]. Five emotions, anger, hate, fear, understanding, and affection, toward the outgroup were measured, as well as conciliatory and aggressive attitudes towards the conflict. The main findings were that positive emotions toward the outgroup mediate the link between originality and positive attitudes toward the conflict [100]. 

To summarize, given that both creativity and reappraisal involve increased activity in the mPFC and given the connection found between the two, it seems that creativity may influence outgroup-targeted emotions involved in intergroup conflict. This effect is accomplished by reappraising perceptions of the outgroup and the conflict, which in turn may lead to experiencing remote emotions that differ from the automatic emotions that usually arise (see Figure 1).

## 7. Ingroup Empathy Biases

Another behavior repeatedly found to be related to intergroup relations is empathy. Empathy refers to the ability to share the emotions of others [101,102] and plays an important role in intergroup relations. Researchers have suggested that empathic reactions may be biased such that they are experienced more intensely toward ingroup members than toward outgroup members [103,104]. It has increasingly been acknowledged that empathy is a crucial factor in determining the course of conflicts [105]. For example, one study examined the effect of social categorization on empathy for pain in the context of the Israeli–Palestinian conflict. The findings showed that when the group membership of the target was explicitly primed, both Israelis and Palestinians rated the pain experienced by their own group members as higher [106]. In addition, empathy can be harnessed for resolving conflict as it fosters reconciliation tendencies toward the outgroup [107,108,109]. For example, a study conducted in post-conflict settings in Northern Ireland [57] found that empathy is positively linked to forgiving the outgroup. Moreover, a series of studies suggests that empathy induction can promote positive attitudes and decrease prejudice toward the outgroup (for a review, see [7]). Evidence for the effect of empathy on attitudes was also found in the context of the Israeli–Palestinian conflict. Jews’ empathy for Palestinians was associated with a decrease in aggressive attitudes toward the Palestinians [109]. 

Perspective taking, one of the components of cognitive empathy, refers to the ability to engage in cognitive processing such that we adopt the perspective of others and see the situation from their point of view [110]. Research has shown that taking another’s perspective makes social interactions smoother, more coordinated, and synchronized [111,112,113]. Accordingly, perspective taking helps alleviate intergroup tensions [114] and increases interpersonal liking between group representatives in intergroup conflicts [115]. Many studies in the field of conflict resolution assume that conflict escalation may partly be explained by ignoring the consequences of the conflict for the other side and focusing on the perspectives and goals of the individual’s own side (e.g., [116,117]). This suggests that perspective taking may help improve conflictual relations and support conflict de-escalation, as was previously shown in different studies (e.g., [118,119,120]). In the context of the Israeli–Palestinian conflict, a study of Jewish participants conducted in 2014 during the Israeli military operation in Gaza found that taking the Palestinians’ perspective predicted mutual forgiveness [5].

Perspective taking involves the mentalizing network, which includes the mPFC, the superior temporal sulcus (STS), the TPJ, and the temporal poles (TP) [121,122]. Intergroup conflict may enhance neural activity related to perspective taking when viewing the suffering of ingroup members. The mPFC was shown to react more to the distress of ingroup versus outgroup members [123], indicating that individuals are more engaged in mental reasoning for ingroup as opposed to outgroup members. In this study, higher activation was found in the mPFC of Black participants when viewing Black victims of a hurricane than when viewing Caucasian victims. Several additional studies showed greater activations in the TPJ when participants viewed photos of ingroup members experiencing emotional pain as compared to outgroup members [124,125,126]. In an attempt to shed some light on the causal role the TPJ plays in behaviors toward ingroup and outgroup members, a TMS study found that inhibitory low-frequency repetitive TMS (rTMS) applied to the right TPJ, but not to the left TPJ, diminished the effect of group membership on the decision to punish. Participants who received rTMS to the right TPJ chose a similar treatment for both ingroup and outgroup members, violating same group social norms. This effect was not observed for the left TPJ or during the sham condition [127], highlighting the importance of the right TPJ in differentiating between ingroup and outgroup in the context of perspective taking.

Like creativity, perspective taking requires flexible shifts between one’s own perspectives and those of another person, inhibition of one’s own emotional state, and online processing of multiple information streams [128]. Indeed, several studies have demonstrated that empathy is associated with certain forms of creativity (e.g., [129,130,131,132]). For example, patients with lesions in the DLPFC exhibited deficits in both empathy and cognitive flexibility [133,134]. In line with this, perpetrators of intimate partner violence were found to exhibit poor cognitive flexibility [135], poor recognition of emotions or thoughts [136], and lower affective empathy [137]. Romero-Martínez et al. [138] suggested that perpetrators of intimate partner violence have a reduced ability to learn from their mistakes, which may be the result of rigid thinking [139]. Additionally, research among children has demonstrated enhanced empathy following creative activities [140], and musical group interactions in children were shown to enhance emotional empathy [141]. Furthermore, in adults, a significant positive correlation was found between empathy and self-reported everyday creativity [142]. 

In summary, in addition to the brain regions shared by creativity, emotion regulation, and stereotyping, common brain regions are also activated by creativity and empathy. Increased activity in the TPJ and the mPFC underlies both perspective taking and creativity (see Figure 1). Additionally, creativity may facilitate the ability to flexibly shift one’s perspective toward that of the outgroup’s viewpoint.

## 8. Herding

Herding is a natural phenomenon often found in animals, in which group members align their emotions, cognitions, and behaviors with those of the group [143,144]. Fish, birds, and ants move jointly with their group in a synchronized and collective manner [145]. In humans, this type of alignment occurs daily, for instance, while clapping together at a performance, feeling the same emotions experienced by people around us, or confirming other people’s opinions and judgments. Research has shown that individuals in a group prefer to synchronize and share their emotions with their ingroup members rather than with those belonging to the outgroup [146,147]. Emotion sharing is positively correlated with the perception of closeness [148]. Thus, experiencing emotions similar to those of an outgroup may have the ability to facilitate likeability and closeness between the groups, thus leading to more positive attitudes. Indeed, when an emotional experience is shared with the outgroup, people’s attitudes change. For example, McDonald et al. [149] manipulated the similarity of emotional experiences of Israeli-Jewish participants and Palestinian-Arab participants. The researchers showed that when the emotion experienced is identical to that experienced by the outgroup; participants rated their conciliatory attitudes as higher. Thus, increased herding with an outgroup has the potential to benefit the relationship between the sides.

Although herding has many advantages [150,151], herding with ingroup members can have a negative effect on intergroup relations. One experiment found that compared to conditions of no mimicking, the mimicking of nonverbal behavior leads to conformity and stereotypes [152]. In addition, in a recent study, participants were assigned to groups of six with two subgroups of three members each, and their subgroup bonding was manipulated. The manipulation entailed wearing the same color T-shirts together with a four-minute chat in which participants from the same group introduced themselves and attempted to find three things they had in common. Synchrony with the ingroup was measured using inter-brain synchrony, i.e., synchronized brain activity during social interactions. A positive relationship was found between ingroup synchrony and hostility toward the outgroup only after the group bonding manipulation and not after the no-bonding control condition [153]. Therefore, less herding with ingroups may result in less inter-group hostility. 

Aligning with another individual requires observing her/his behavior, activating the behavior’s mental representation, and executing it. Therefore, brain areas that underlie the observation–execution (OE) system, such as the IFG [154], are part of the neural correlates of herding [144]. For example, increased activation in the IFG was found during verbal and nonverbal synchronization [155,156]. As mentioned earlier, this brain region is also associated with the evaluation phase in creative performance [26,34,35]. Thus, although the IFG is part of the ECN system, it is also associated with the OE system. It seems that increased activity in the IFG is responsible for imitating an ingroup member (the OE system). Therefore, during stereotyping, the OE system may be responsible for conforming with the group stereotype as well as monitoring to ensure that the stereotype is similar to that of the ingroup. Hence, decreased activity in the IFG is associated with less herding, less strict monitoring, and more creativity.

Because herding is a phenomenon in which individuals follow the ingroup’s cognition and ideas about an outgroup member [144,157], it leaves these individuals with unoriginal ideas and fixedness of thought. Individuals with a strong tendency to align with others may be less flexible and less open to experiencing new situations. Indeed, early research suggests that creativity and conformity are directly opposed [158]. Along the same line, it was found that openness to experience is negatively correlated with conformity [159]. In addition, original individuals think in a remote and non-standard way that requires constrained herding with the ingroup. Furthermore, although some research suggests that individuals with autism spectrum disorder are less creative [160], these individuals were found to generate more creative and novel metaphors compared to controls [161,162]. They also exhibit impairment in aligning with the group [163].

Hence, it is possible that creativity can also diminish herding with the ingroup, thus decreasing hostility and promoting peace in a conflict (see Figure 1). In addition, decreasing activity in the IFG also diminishes the process of observing and executing the behavior of others, thus leaving more space for free thinking. 

## 9. Creativity-Based Interventions for Conflict Resolution

Previous studies have shown that creativity is trainable. Scott et al. [164] analyzed 70 creativity training studies and concluded that creativity training does enhance creative performance. This training should be based on the cognitive abilities underlying creative efforts. It should be long and challenging, based on contextual approaches, and include a series of exercises [164]. Moreover, idea production and cognitive training have been proven to be the most effective types of creativity training, while some commonly applied training strategies, specifically imagery training, have been proven to be less effective [165]. Accordingly, we recommend future studies to investigate different types of creativity training and their effects on conflict-related biases. For example, a study of a series of training sessions versus a single session. In such studies, participants could perform divergent thinking tasks [166], such as the alternative uses task in which participants are asked to give uncommon uses to different objects every day for two weeks or in a single session and compare their emotions and biases toward the outgroup with control groups and with each other.

Another example is the study of group creativity which involves both creativity and cooperation. Notably, much creativity emerges in social settings in which interactions with other people may contribute to the creative outcome [167]. This suggests that communication between group members may substantially affect creativity [168]. Group creativity training may result in more creative performance than individual creativity training and thus may be more effective in overcoming biases. For example, Xue et al. [169] investigated group creativity performance in different types of dyads. They found that the performance of two less creative individuals was as good as that of two highly creative individuals during a group creativity task. Moreover, improvement in group creativity emerged following cooperation [170] as opposed to competition (e.g., [171]). Thus, in future research, it is important to study the differences between individual creativity training and group creativity training as well as to compare training consisting of a number of sessions versus a single session. The insights emerging from such an investigation can eventually be translated into effective interventions in educational programs for reducing biases and conflicts between groups.

## 10. Limitations and Challenges

Although the review of the literature discussed above seems promising, some limitations should be considered. First, the current article is a non-systematic narrative review, and thus, it does not cover all of the publications related to creativity. We suggest that after collecting enough evidence on the proposed model, a systematic review will be more suitable. In addition, we acknowledge that, as there is no direct empirical evidence demonstrating the effect of creativity on cognitive-related biases, the current manuscript focuses on indirect evidence linking these concepts. We offer here a new approach for future studies to examine these links and provide more concrete evidence.

In addition to the limitations of this study, there is some evidence that contradicts our proposed model and constitutes a challenge. First, creativity also has a dark side, namely malevolent creativity, which is the use of creativity for the purpose of harming the self or others. Malevolent creativity involves both originality and damage [172]. Creativity may also be linked to criminal actions and anti-social behaviors [173,174]. 

Second, although the same brain areas underlie both creativity and intergroup biases, we cannot conclude from this about the casual relationship between them. It would be interesting to investigate the casual relationship in future studies using neurostimulation methods. Third, the increased activity in the IFG was also found to underlie emotional empathy, which refers to the ability to relate to other people’s emotions [101,175,176]. While one might expect that increased empathy would enhance prosocial behaviors, Bloom [177] recently suggested that emotional empathy actually has a negative effect on decision-making, particularly in the context of moral decisions. In his book *Against Empathy*, Bloom [178] contends that empathy, specifically emotional empathy, is one of the most profound yet overlooked sources of human conflict. He argues that empathy is used by those who wish to generate hatred toward outgroups and to gain support for war. For instance, in order to evoke hatred toward immigrants, politicians create and highlight narratives about the victims of crimes committed by individual immigrants [178]. Likewise, Buffone and Poulin [179] showed that when participants were motivated to feel empathy for a student who was financially in need, they were more prone to administer a larger dose of hot sauce to the student’s innocent competitor in a competition for a cash prize. These findings support the view that increased IFG activity is not only related to reduced creativity [180] but is also related to increased emotional empathy. Future studies on creativity are needed in order to investigate the conditions and circumstances in which creativity is beneficial to intergroup relations in the context of conflict.

Finally, the motivation of people to experience different types of emotions varies depending on the context [181]. For example, during conflicts, people are motivated to experience negative emotions and avoid experiencing positive ones [182]. An intervention aimed at increasing the positive emotions and decreasing the negative emotions might have the opposite effect if it is not in the same line with the motivation [183]. This might suggest that indirect interventions may be more beneficial in affecting conflict-related biases. Future research could shed light on this issue and examine whether creativity intervention affects emotional experiences during conflict in an indirect way and what might overcome the obstacle of motivation.

## 11. Conclusions

**Figure 2:**
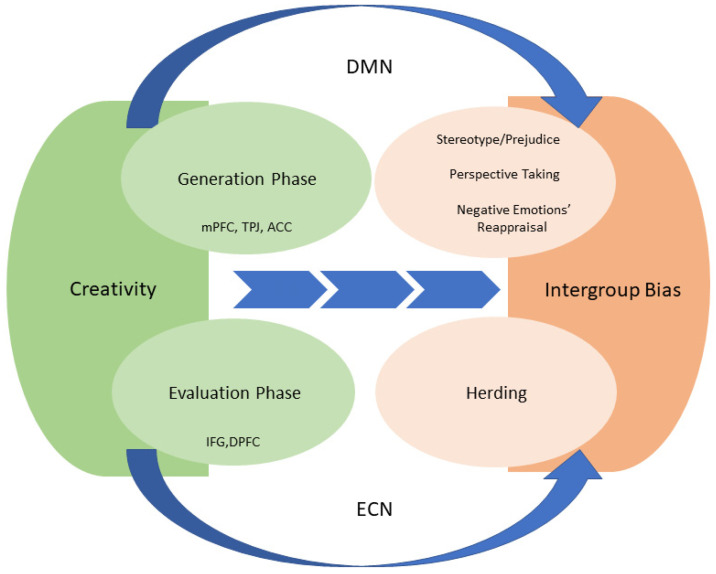
The underlying mechanism of creativity as an intervention for peace. According to our model, increased creative thinking involves the generation phase comprising the mPFC and TPJ. This phase is linked to enhanced reappraisal and perspective taking and to reduced stereotypes and prejudice. Increased creative thinking also involves the evaluation phase comprising the IFG. This phase is linked to reduced herding.

This literature review of creativity and conflict-related biases offers an integrative framework for understanding the mechanisms underlying a potential intervention for reconciliation that is based on creativity. We demonstrate how the components of creative cognition (flexibility, originality) are related to conflict-related cognitions and rely on shared networks. We describe the twofold model of creativity that involves a generation phase and an evaluation phase. The generation phase is marked by increased activation of the DMN, which includes the mPFC and the TPJ (see Figure 2). The mPFC is also related to the emotional regulation of negative emotions, thus reducing negative outgroup-targeted emotions. Increased activity in the TPJ and the mPFC involves perspective taking. Finally, increased activity in the mPFC also reduces stereotypes and prejudice. The evaluation phase of creativity involves the deactivation of the IFG, which results in behavior that is less aligned with the ingroup. Thus, creativity training has the potential to influence biases, enhance the ability to experience different emotions that are not automatic, take the outgroup perspective, and broaden the narrow thinking style of stereotyping. All of these may help overcome the functional fixedness that characterizes the conflict mindset, thus freeing the mind to consider more viewpoints.
